# Supplementary material for: Longitudinal Metabolomic Analysis Reveals Gut Microbial-Derived Metabolites Related to Formula Feeding and Milk Sensitization Development in Infancy
Source: Metabolites. 2022 Jan 28;12(2):127. doi: 10.3390/metabo12020127 (PMC8877196; doi:10.3390/metabo12020127)
Supplement: Supplementary file 1 [file metabolites-12-00127-s001.zip › metabolites-1549505-supplementary.pdf]

Supporting Information

Longitudinal Metabolomic Analysis Reveals Gut Microbial-Derived Metabolites Related to Formula Feeding and Milk Sensitization Development in Infancy

Ching-Min Tang, Gigin Lin, Meng-Han Chiang, Kuo-Wei Yeh, Jing-Long Huang, Kuan-Wen Su, Ming-Han Tsai, Man-Chin Hua, Sui-Ling Liao, Shen-Hao Lai, and Chih-Yung Chiu*

Supplementary Figures and Tables

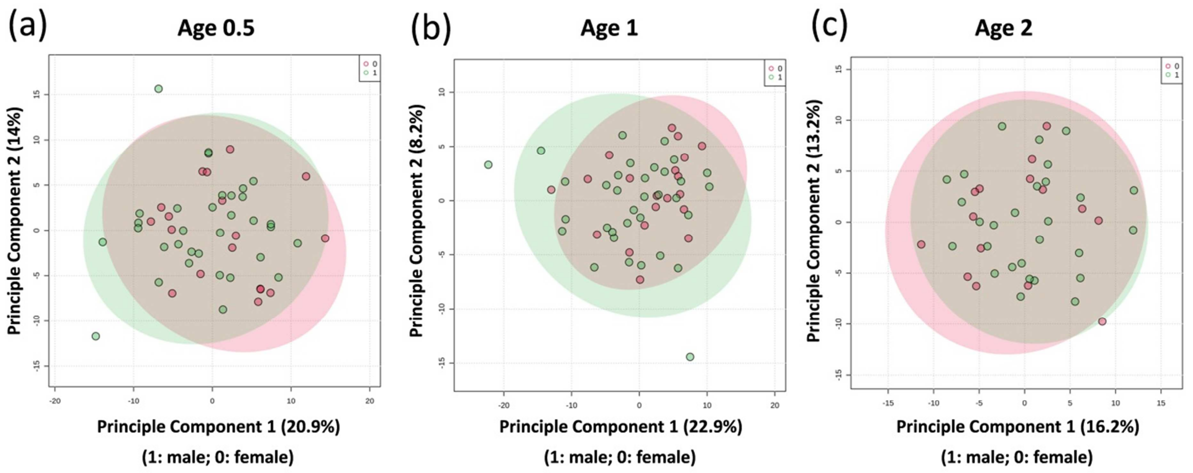

Figure S1. PCA score plots from the analysis of urine ^{1}H-NMR spectra between children in different sex at age 0.5 (a), age 1 (b), and age 2 (c).

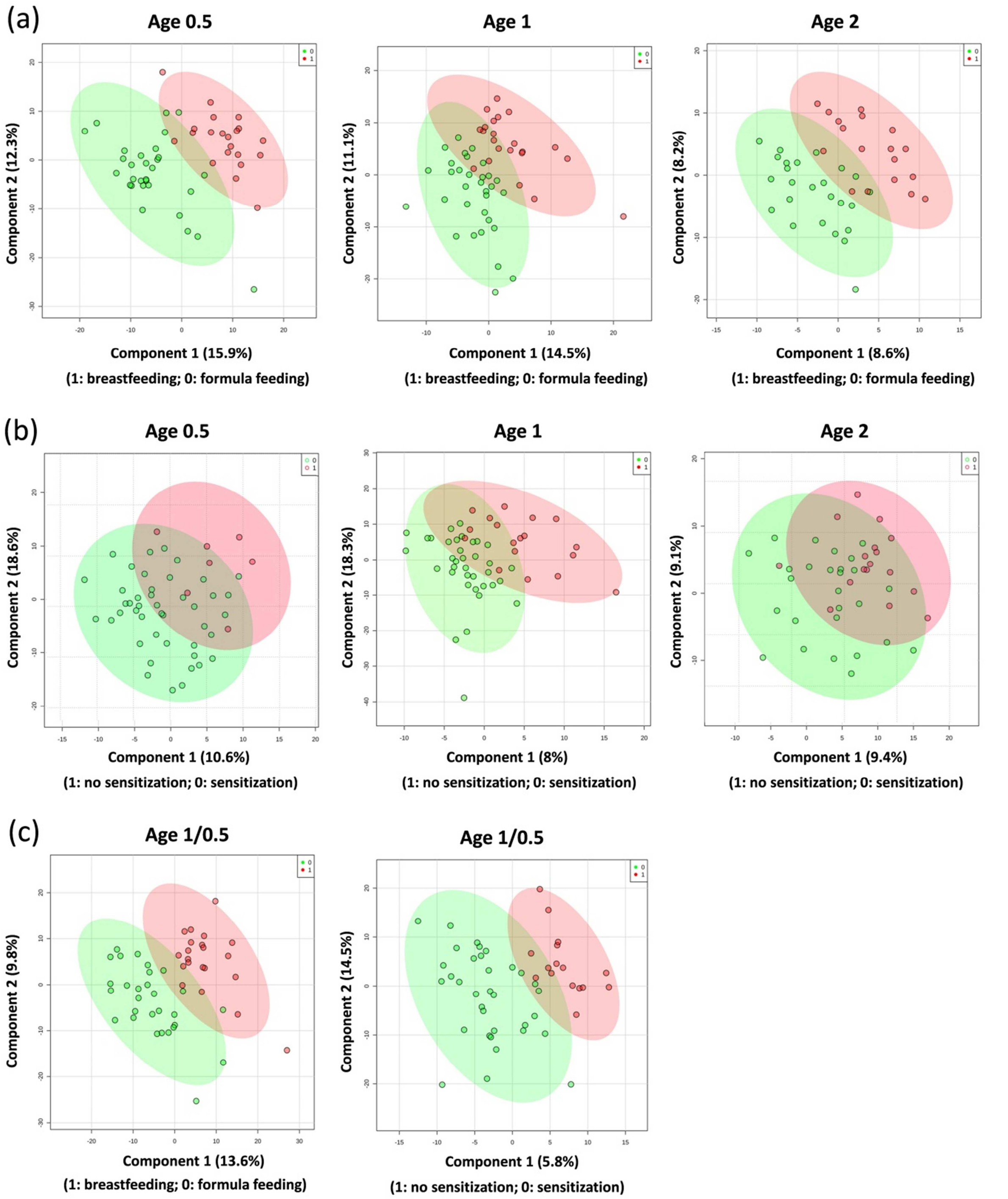

Figure S2. PLS-DA score plots from the analysis of urine ^{1}H-NMR spectra among children in different breastfeeding patterns (a), with and without milk sensitization (b) at age 0.5, 1, and 2 and the comparison from 6 months to 1 year of age (1y/6m) (c).

Table S1. PLS-DA parameters and permutation test for distinguishing between different breastfeeding patterns and milk sensitization.

	Formula feeding					Milk sensitization				
	PLS-DA parameters					PLS-DA parameters				
Age (yr)	Components[a]	Q^2	R^2	Q^2/ R^2	$P_{permutation}$[b]	Components[a]	Q^2	R^2	Q^2/ R^2	$P_{permutation}$[b]
0.5	1	0.40	0.57	0.70	0.453	1	-0.33	0.34	-1.00	0.743
1	1	-0.32	0.32	-1.02	0.688	1	-0.19	0.45	-0.41	0.670
2	1	-0.34	0.44	-0.77	0.692	1	-0.49	0.46	-1.06	0.997
1/0.5	2	0.31	0.76	0.40	0.496	2	0.02	0.70	0.03	0.633

PLS-DA, partial least squares-discriminant analysis; yr, year; Q^2, predictive capability; R^2, correlation coefficients; [a]The number of components based on Q^2 indicates the best classifier of PLS-DA using a 10-fold cross-validation method. [b]1000 random permutations were performed.

Table S2. The VIP score and fold change of metabolites significantly differentially expressed between exclusive breastfeeding and formula feeding at different years of age.

Metabolites	Chemical shift, ppm (multiplicity[a])	Age 0.5			Age 1			Age 2		
		VIP score[b]	Fold change[c]	*p*	VIP score	Fold change	*P*	VIP score	Fold change	*p*
Glutarate	1.76-1.80(tt)	1.96	1.48	**<0.001**	0.73	0.94	0.276	0.13	1.03	0.868
3-Methyl-2-oxovaleric acid	1.10-1.11(d)	2.22	1.64	**<0.001**	0.07	0.98	0.935	0.47	0.89	0.600
Lysine	1.89-1.91(m)	1.54	1.33	**<0.001**	0.78	0.94	0.243	0.15	0.98	0.842
Threonine	4.25-4.27(d)	1.40	1.27	**<0.001**	0.08	1.01	0.918	0.02	1.01	0.978
N-Phenylacetylglycine	7.41-7.45(s)	2.53	2.03	**<0.001**	0.18	0.94	0.872	0.70	1.08	0.489
3-Indoxysulfate	7.50-7.52(d)	2.68	2.47	**<0.001**	1.24	1.09	0.318	0.16	0.99	0.874
Hippuric acid	7.62-7.66(tt)	2.43	2.36	**<0.001**	1.01	1.26	0.475	0.71	0.91	0.588
N,N-Dimethylglycine	2.93-2.93(s)	1.85	0.61	**<0.001**	0.26	0.97	0.781	1.54	0.85	0.064
Creatine	3.93-3.94(s)	2.03	1.64	**<0.001**	1.34	1.24	0.243	0.26	1.07	0.804
N-Acetyltyrosine	7.16-7.18(d)	2.07	1.90	**<0.001**	1.19	1.20	0.284	1.62	1.30	0.121
Propylene glycol	1.14-1.15(d)	2.15	0.37	**<0.001**	1.28	0.73	0.298	0.44	0.83	0.745
Pantothenate	0.93-0.94(d)	1.06	1.21	**<0.001**	0.31	0.97	0.669	0.00	1.01	1.000
Galactose	4.58-4.61(d)	1.45	1.42	**<0.001**	0.52	1.07	0.589	0.87	1.09	0.386
Fucose	1.24-1.25(d)	1.38	0.68	**<0.001**	0.90	0.91	0.288	0.88	0.90	0.288
2-Oxoglutaric acid	3.00-3.01(t)	1.40	0.66	**0.001**	0.05	1.00	0.952	0.88	0.93	0.238
2-Hydroxyisobutyric acid	1.35-1.37(s)	0.89	1.15	**0.001**	0.36	0.98	0.517	0.16	0.98	0.815
Valine	1.04-1.05(d)	0.93	1.16	**0.001**	0.07	1.01	0.909	0.51	0.95	0.507
Dimethyl sulfone	3.15-3.16(s)	1.08	1.27	**0.004**	0.12	1.00	0.884	0.17	1.02	0.828
Allantoin	5.39-5.40(s)	1.53	1.46	**0.005**	2.00	0.76	0.072	0.01	0.97	0.995
1-Methylnicotinamide	9.27-9.29(s)	1.19	1.35	**0.015**	0.18	0.97	0.878	0.47	1.15	0.696
Adipate	1.55-1.56(m)	0.81	1.18	**0.035**	1.21	0.89	0.126	0.46	0.97	0.594
Dimethylamine	2.71-2.73(s)	0.49	1.07	**0.027**	1.32	0.90	**0.036**	0.68	1.07	0.375

[a]Multiplicity, tt, triplet of triplets; d, doublet; m, multiplet; s, singlet; t, triplet. One of the proton assignments of the metabolite without signal overlapping was selected and the integral of selected peak was presented as a range of chemical shift. [b]VIP score were obtained from PLS-DA. [c]Fold change was calculated by dividing the value of metabolites in children receiving formula feeding by exclusive breastfeeding. All FDR-adjusted *p* values < 0.05, which is in bold, are significant. VIP, Variable Importance in Projection.

Table S3. The VIP score and fold change of metabolites significantly differentially expressed between with and without milk sensitization at different years of age.

Metabolites	Chemical shift, ppm (multiplicity[a])	Age 0.5			Age 1			Age 2		
		VIP score[b]	Fold change[c]	*p*	VIP score	Fold change	*p*	VIP score	Fold change	*p*
2-Oxoglutaric acid	3.00-3.01(t)	1.92	0.74	**0.035**	1.16	1.20	0.103	1.84	1.25	**0.007**
Acetone	2.23-2.24(s)	2.13	0.67	**0.028**	1.58	1.20	**0.025**	0.58	1.26	0.508
3-Methyl-2-oxovaleric acid	1.10-1.11(d)	1.02	1.12	0.223	2.01	0.79	**0.006**	0.46	0.89	0.581
Pantothenate	0.93-0.94(d)	0.27	1.04	0.687	1.62	0.86	**0.009**	1.05	0.89	0.114
N-Phenylacetylglycine	7.41-7.45(s)	1.34	1.15	0.252	2.53	0.67	**0.010**	1.55	0.82	0.096
Glutarate	1.76-1.80(tt)	0.75	1.08	0.304	1.40	0.89	**0.016**	0.22	1.01	0.767
Allantoin	5.39-5.40(s)	0.63	1.07	0.584	2.29	0.76	**0.019**	1.19	0.84	0.216
Formic acid	8.45-8.47(s)	0.21	1.13	0.859	2.45	1.28	**0.020**	1.59	1.29	0.138
Lysine	1.89-1.91(m)	0.52	1.05	0.432	1.29	0.90	**0.026**	0.26	0.99	0.707
3-Indoxysulfate	7.50-7.52(d)	1.29	1.08	0.309	2.20	0.75	**0.043**	0.84	0.91	0.370

[a]Multiplicity, s, singlet; d, doublet; m, multiplet; t, triplet; tt, triplet of triplets. One of the proton assignments of the metabolite without signal overlapping was selected and the integral of selected peak was presented as a range of chemical shift. [b]VIP score were obtained from PLS-DA. [c]Fold change was calculated by dividing the value of metabolites in children with by without milk sensitization. All FDR-adjusted *p* values < 0.05, which is in bold, are significant. VIP, Variable Importance in Projection.
